# Reply to: Comments on “Finding the optimal mammography screening strategy: A cost‐effectiveness analysis of 920 modeled strategies”

**DOI:** 10.1002/ijc.34042

**Published:** 2022-05-06

**Authors:** Lindy M. Kregting, Valérie D. V. Sankatsing, Eveline A. M. Heijnsdijk, Harry J. de Koning, Nicolien T. van Ravesteyn

**Affiliations:** ^1^ Department of Public Health, Erasmus MC University Medical Center Rotterdam Rotterdam The Netherlands

**Abbreviations**
CEAs: cost‐effectiveness analyses
DCIS: ductal carcinoma in situ
MISCAN: microsimulation screening analysis
NNI: number needed to invite
NNS: number needed to screen
QALYs: quality adjusted life years
RCTs: randomized controlled trials.

Dear editor,

In a letter, Dr. Alain Braillon criticized the methodology of our study “Finding the optimal mammography screening strategy: a cost‐effectiveness analysis of 920 modeled strategies.” With this letter, we aim to reply to the points raised and emphasize the validity of our model and why we believe modeling studies like ours are valuable to screening policy making.

Braillon unfortunately disregards the enormous efforts international modeling groups have made and continue to make, to obtain best estimates of the natural history of breast cancer using high quality data from randomized controlled trials (RCTs) and observational studies. His main criticism is that the model is too optimistic by overestimating benefits and underestimating harms.

With regard to the benefits, some points that are raised are based on incorrect interpretation of our inputs. For example, his statement that “the model assumed a life expectancy of 100 year” is not true. In our study we used Dutch lifetables representing the actual life expectancy of Dutch women with a maximum of 100 years. Setting a maximum of 100 years means that if this had any effect on the benefits, it will lead to a slight underestimation. In addition, we agree that there have been important improvements in screening as well as treatment (mainly adjuvant therapy), and those improvements are incorporated in the Microsimulation Screening Analysis (MISCAN) Breast model. A previous study by de Gelder et al showed that in a situation with adjuvant therapy and screening, adjuvant therapy was responsible for a mortality reduction of 33% and screening was responsible for a mortality reduction of 21% compared to a situation without adjuvant therapy and screening.^1^ Even though the contribution of adjuvant therapy is larger, the contribution of screening remains substantial. Furthermore, Braillon questions the use of 100% attendance assumptions in cost‐effectiveness analyses (CEAs). However, this is a standard assumption in CEAs to investigate the maximal potential of strategies and to allow for comparison of these strategies within the study and between studies. In order to see the effects of different strategies with more realistic attendance rates, we performed sensitivity analyses in which we used observed age‐specific attendance rates from the Netherlands with an average attendance of 76% for the current Dutch breast cancer screening strategy. However, this did not lead to big changes in the screening strategies present on the efficiency frontier. Finally, Braillon used our data to calculate the number needed to invite (NNI) and found it too low compared to a Norwegian study. However, in order to compare the model predicted NNI and number needed to screen (NNS) to previously reported estimates, it is necessary to use undiscounted model results. In an undiscounted model run with biennial screening for the ages 50 to 74 using Dutch age‐dependent attendance rates, we find an NNI of 100 and an NNS of 76. This comes close to the NNS of 111 to 143 calculated by Paci et al for biennial screening for the ages 50 to 69 using all available evidence for Europe.^2^ The remaining difference can be explained by additionally screening women aged 70 to 74 years, in which breast cancer incidence is higher and consequently more breast cancer deaths can be prevented.

With regard to the harms, our article included an analysis of multiple harms of screening. An often discussed harm of breast cancer screening is overdiagnosis (ie, the diagnosis of cancers with screening that would not have led to complaints if left undiagnosed). Our study found that in the case of biennial screening for the ages 50 to 74, 5 breast cancers would be overdiagnosed on a total of 154 breast cancer cases of which 73 were screen‐detected per 1000 screened women. This means that 1 in 14 screen detected breast cancers (7%) was overdiagnosed, which is again well in line with Paci et al, and also in agreement with Bulliard et al who state that estimates of overdiagnoses higher than 10% are likely to derive from inaccuracies in the study design.^2^, ^3^ All harms analyzed in the study led to a subtraction of a harm specific disutility from the normative utility assigned to the simulated women. This included disutilities for participating in screening, referral after a positive screening result, and overdiagnosis.^4^ Thus, by presenting quality adjusted life years (QALYs) all screening‐related benefits and harms are taken into account in our study.

Even though model calculations require some assumptions, most models are extensively calibrated and regularly validated on RCTs and observational data. Using these models, best estimates of the natural history of diseases can be retrieved which would not have been possible or ethical to find in trials or observational studies. Such modeling has for instance helped to disentangle the contribution of screening vs treatment on the observed decline in breast cancer mortality, narrow the uncertainties around ductal carcinoma in situ (DCIS), and partially influenced criteria for inclusion in DCIS treatment trials.^5^ Our article shows such well‐thought modeling efforts are more efficient than starting 920 small scale trials to try and give answers to find an optimal screening scenario. We show that without increasing the number of screens, we can improve upon the current screening strategy by moving to triennial screening for the ages 44 to 71, or 44 to 74, which is predicted to increase benefits and reduce costs compared to the current screening strategy. We do agree with Braillon that evaluating risk‐based screening strategies is a crucial next step for further improving breast cancer screening in the future.

## CONFLICT OF INTEREST

The authors declare no conflict of interest.

## AUTHOR CONTRIBUTIONS

Lindy M. Kregting: Conceptualization, formal analysis, writing ‐ original draft, writing ‐ review & editing. Valérie D. V. Sankatsing: writing ‐ review & editing. Eveline A. M. Heijnsdijk: writing ‐ review & editing. Harry J. de Koning: Conceptualization, writing ‐ review & editing. Nicolien T. van Ravesteyn: Conceptualization, supervision, writing ‐ review & editing. The work reported in the article has been performed by the authors, unless clearly specified in the text.

## Data Availability

Data used as input for the MISCAN‐Breast model can be requested from the primary source as stated in the supplementary material of the original article (DOI: 10.1002/ijc.34000; Table S1). Model outcome data can be made available upon reasonable request.
